# Integrated Transcriptomic and Metabolomic Analysis Reveals Possible Molecular Mechanisms of Leaf Growth and Development in *Disanthus cercidifolius* var. *longipes*

**DOI:** 10.3390/metabo14120654

**Published:** 2024-11-25

**Authors:** Xiaoming Tian, Guangfeng Xiang, Cun Mou, Lu Zhu, Jing Song, Gaofei Li, Hao Lv

**Affiliations:** 1 Institute of Plant Conservation, Hunan Botanical Garden, Changsha 411006, China; iceenergy@163.com (G.X.); stevemoon@126.com (C.M.); jy01200125zl@163.com (L.Z.); lgf02@163.com (G.L.); 2 Forestry Affairs Center of Hunan Province, Changsha 410114, China

**Keywords:** *Disanthus cercidifolius* var. *longipes*, photosynthesis, flavonoid synthesis, anthocyanin synthesis

## Abstract

**Background:** *Disanthus cercidifolius* var. *longipes* is an ancient relic plant unique to China. However, the typical shade-loving plant is largely exposed to the sun, which poses a major challenge to its conservation. **Methods:** This study explored dynamic changes in primary and secondary metabolites in *D. cercidifolius* leaves at different stages of development, combining metabolomics and transcriptome analysis to discuss the differentially accumulated metabolites (DAMs) and differentially expressed genes (DEGs). **Results:** The DAMs and DEGs were enriched in pathways related to photosynthesis, carbon (C) metabolism, anthocyanin synthesis, plant hormone signal transduction, and flavonoid synthesis. At the initial stage of leaf development, many primary metabolites were synthesized in the leaves. Before leaf maturity, many primary metabolites were converted into secondary metabolites. Combined transcriptome and metabolome analysis showed that the metabolites and genes related to anthocyanin synthesis and flavonoid metabolism were upregulated. In contrast, the genes related to C metabolism and C fixation were downregulated. After leaf maturity, photosynthetic capacity increased, total flavonoid content peaked (implying the strongest photoprotection capacity), and the transformation of anthocyanins and flavonoids was weakened. **Conclusions:** Light intensity indirectly affects the accumulation of the primary and secondary metabolism of *D. cercidifolius*. With the enhancement of photoprotection, the photosynthetic energy capacity decreases. It is, therefore, inferable that *D. cercidifolius* has shading properties and achieves a stable nutrient supply during growth and development through these strategies. Thus, *D. cercidifolius* protection requires a shaded environment.

## 1. Introduction

*Disanthus cercidifolius maxim* var. *longipes* H.T. Chang is a deciduous shrub in the family Hamamelidaceae, an ancient relic plant endemic to China [1]. Hamamelidaceae is one of the ancient plant groups. As the most primitive single species genus in the family, the genus *Disanthus Maxim* is of great significance in the study of the reproductive strategies of ancient plants. *D. cercidifolius* is native to Japan, and *D. cercidifolius* var. *longipes* is distributed in China. In addition, *D. cercidifolius* var. *longipes* is considered the equivalent of *Disanthus ceriolius* [2]; therefore, *D. cercidifolius* var. *longipes* is of great significance in the biogeographic study of plant transmission in East Asia. Its distribution area is narrow, and the number of remnants is relatively low. It is only sporadically distributed in small parts of the Provinces of Hunan, Jiangxi, and Zhejiang, China [2]. It has been listed in the World Conservation Union Red List, and the protection level is endangered (EN) [3]. *D. cercidifolius* has an elegant posture, is heart-shaped, and is composed and richly colored leaves and deciduous and double flowers, with high ornamental and garden art value. Researchers have focused on its conservation and domestication to expand its population and enhance its economic value.

The current *D. cercidifolius* habitat does not offer optimal conditions for its growth and development, which has hampered the stable growth of its population [1]. Horton et al. [4] classified plants into three types according to light intensity requirements: sunny, shaded, and intermediate. *D. cercidifolius* is a typical shade-loving plant [5,6]. *D. cercidifolius* has been observed to dominate the shrub layer in both mixed coniferous broad-leaved and evergreen broad-leaved forests [7]; this may be due to the destruction of vegetation caused by increased human activities. According to Chen et al. [8], light availability is the major factor causing seedling death. Therefore, to better protect the species in the future, the physiological activities of the leaves of seedlings should be investigated.

Photosynthesis is the basic energy conversion process in plants. Light is the basic driving force and an important stress factor of photosynthesis. Different light intensities can affect the accumulation of plant metabolites, and secondary metabolites play a key role in the plant’s response to environmental changes [9]. When the environment around the plant changes, the plant’s metabolic pathways will also change, causing changes in the type and content of metabolic products. Plant leaves fix carbon (C) through photosynthesis and transport it to various organs through photosynthetic products to support various physiological activities [8]. Photosynthetic capacity varies across different stages of leaf development, and the physiological activities of leaves are also different, which implies that the gene expression and metabolite profiles of leaves at different stages are distinct [10,11,12,13]. Consequently, conservation practitioners may need to adopt different strategies at different stages of leaf development. However, there is a gap in our understanding of the leaf development processes in *D. cercidifolius*, which hampers the adoption of such conservation strategies.

Recently, numerous scholars have explored various aspects of *D. cercidifolius* biology, primarily focusing on seed characteristics, breeding technologies, population structure, and floristic composition, among other aspects [2,13,14,15]. A stable development sequence of the leaves is key to the normal growth, development, and successful reproduction of *D. cercidifolius*. To our knowledge, no systematic studies have explored this species’ metabolite profiling and molecular mechanisms of leaf development. Leaf age directly affects light interception and the C sequestration capacity of leaves, and such indicators are critical for the growth and development of plants [16]. In the present study, *D. cercidifolius* leaves at different developmental stages were selected to explore the dynamic changes in their primary and secondary metabolites. Differentially accumulated metabolites (DAMs) and differentially expressed genes (DEGs) during leaf growth and development were explored via metabolome and transcriptome analyses to reveal metabolite accumulation principles. The present study’s findings could provide insights that could facilitate the conservation of the endangered *D. cercidifolius*.

## 2. Materials and Methods

### 2.1. Plants

The experimental site was located at a nursery of the Hunan Botanical Garden (113° E, 28°20′ N), under a subtropical monsoon climate, with an average annual temperature of 17.2 °C and an average relative humidity of 80%. The soil is quaternary reticulated red soil with a pH of 5.6.

The experimental materials were 5-year-old stem cut-off seedlings of *D. cercidifolius* planted in the nursery. On 25 April 2023 (20 d after leaf spreading), 25 May 2023 (50 d after leaf spreading), and 25 June 2023 (80 d after leaf spreading), branches on the sunny side with good growth were selected on the morning when the weather was sunny, and there were no water drops on the leaf surfaces. Counting from the branch’s top, the 1st to 3rd leaves were collected. They were marked as follows: F1, young leaves (leaves unfolded, but the internal structure is not fully mature); F2, near-mature leaves (the leaf area just reaches the maximum after leaf unfolding, and the internal structure of leaves tends to be mature); and F3, mature leaves (leaves are fully mature). The leaves were collected from three trees at different developmental stages, and five branches in the same direction from each tree were selected to collect leaves. After the leaves were collected, they were snap-frozen in liquid nitrogen, and all collected samples were stored at −80 °C for subsequent analyses.

### 2.2. Leaf Age Determination

From June 2021 to December 2022, the growth and development characteristics of *D. cercidifolius* were observed. *D. cercidifolius* is a deciduous shrub. The leaf buds of the long petiole are single buds, including terminal buds and axillary buds. Leaf buds began to differentiate in July, grew rapidly from the end of October to the beginning of November, and then stopped growing and entered dormancy. In the middle of March of the next year, the leaf buds resumed growth, and the leaf buds (top buds) from the end of March to the beginning of April opened and expanded leaves. By the end of June, the leaf buds developed into branches, and all the leaves on the branches matured. New shoots sprouted from axillary buds on the branches from May to July and developed into new branches. In mid-September, the leaf color changed from green to a gradient consisting of green, purple, and orange. The leaf color was red or orange-red in late October, and defoliation occurred. In early November, all the leaves fell. Therefore, there would be leaves with different leaf ages on the same plant from May to October. The leaf buds of *D. cercidifolius* emerge from the end of March to the beginning of April. The leaves after 20 d of leaf bud opening and leaf expansion are recorded as young leaves (F1), the leaves after 50 d of leaf expansion are recorded as near-mature leaves (F2), and the leaves after 80 d of leaf expansion are recorded as mature leaves (F3) to ensure that the measured leaves are the corresponding leaf ages.

### 2.3. Detection of Leaf Chemical Composition

To explore differences in the composition of leaves at each stage, free amino acid, soluble protein, total soluble sugar, total flavonoid [17], total phenol [18], total alkaloid [19], and total triterpene [20] concentrations were determined. The specific methods are as follows:

(a) Free amino acids: After the samples were ground to a powder in liquid nitrogen, approximately 0.1 g was added to 1 mL of 10% acetic acid solution, fully homogenized, and extracted for 15 min in a boiling water bath. The sample was cooled and centrifuged at 8000× *g*, and the supernatant was diluted for testing. In total, 10 μL of the test solution, 120 μL of an acetic acid–sodium acetate buffer solution, 100 μL of a 3% ninhydrin solution, and 10 μL of a 0.3% ascorbic acid solution were added to a tube. After mixing, the tube was sealed and placed in a boiling water bath for 15 min. After cooling, the tube was centrifuged at 8000× *g* for 5 min. The supernatant was used to determine the absorbance (Spectra Max Single Mode Reader ABS Plus, Molecular Devices, Shanghai, China) at 570 nm.
(b) Soluble protein: Approximately 0.05 g of fresh sample was added to 1 mL of distilled water and then homogenized in an ice bath. The resulting sample was centrifuged, and the supernatant was used for testing. In total, 20 μL of the supernatant solution was added to 200 μL of Coomassie brilliant blue G-250 solution and mixed well, and the absorbance at 595 nm was determined.
(c) Total soluble sugar: Approximately 0.05 g of fresh sample was ground into a homogenate, heated and cooled in a water bath, and then brought to a volume of 1.25 mL. After centrifugation, the supernatant was diluted at room temperature (25 °C) to be tested. In total, 40 μL of the supernatant was added to 40 μL of distilled water, 20 μL of an anthrone–sulfuric acid solution, and 200 μL of concentrated sulfuric acid. After mixing well and cooling, 200 μL was used to measure the absorbance at 620 nm.
(d) Total flavonoids: Approximately 0.05 g of fresh sample was added to 1 mL of a 60% ethanol solution. After ultrasonic extraction and centrifugation, the supernatant (diluted) was used for testing. In total, 20 μL of the supernatant was added to 6 μL of 5% sodium nitrite, mixed, and allowed to rest. Next, 6 μL of a 10% Al(NO_3_)_3_ solution was added and mixed well, followed by 80 μL of a 4% NaOH solution and 88 μL of distilled water. Finally, the absorption value at 510 nm was determined.
(e) Total phenols: Approximately 0.1 g of fresh sample was added to 1 mL of 60% ethanol, fully homogenized, and ultrasonicated for 30 min. In total, 10 μL of the resulting supernatant was added to 50 μL of Folin’s reagent, and 50 μL of a 12% Na_2_CO_3_ solution and 90 μL of distilled water were added. The absorbance at 760 nm was determined.
(f) Total alkaloids: Approximately 0.02 g of dry sample was extracted with concentrated ammonia. Next, 1 mL of a chloroform–methanol mixed solution (chloroform/methanol = 4:1) was added. After ultrasonication, the resulting solution was centrifuged for 10 min. In total, 20 μL of the supernatant was dried at 80 °C. Next, 100 μL of a 0.2 mol/L potassium hydrogen phthalate buffer and 80 μL of bromocresol green solution were added. The mixture was shaken vigorously, after which 300 μL of chloroform was added, followed by more shaking. Finally, after 30 min, 200 μL of the lower chloroform layer was used to determine absorbance at 410 nm.
(g) Total triterpenes: Approximately 0.05 g of dry sample was placed into a 2 mL centrifuge tube. In total, 1 mL chloroform was added, ultrasonicated, and centrifuged. In total, 50 μL of the supernatant was steam dried at 60 °C. Next, 20 μL of a 5% vanillin-acetic acid solution was added to dissolve the precipitate, and then 50 μL of perchloric acid was added. The solution was mixed well, incubated in a 60 °C water bath for 15 min, and cooled in an ice bath. Next, 250 μL of glacial acetic acid was mixed and incubated for 15 min. Finally, 200 μL of the resulting supernatant was used to measure the absorbance at 548 nm.

### 2.4. Metabolite Analysis

The components in leaves at different developmental stages were quantified to explore changes in metabolite content during development and consider the difficulty of absolute quantification of rich metabolites. The samples were vacuum freeze-dried in a lyophilizer and then ground (30 Hz, 1.5 min) to a powder using a grinder (MM 400, Retsch). In total, 50 mg of the sample powder was added to 1200 μL of a pre-cooled (−20 °C) 70% methanolic aqueous internal standard extract (samples under 50 mg were added at the rate of 1200 μL extractant per 50 mg sample). Samples were vortexed six times every 30 min for 30 s. After centrifugation (12,000 rpm, 3 min), the supernatant was aspirated, filtered through a microporous membrane (0.22 μm pore size), and then stored in an injection vial for ultra-performance liquid chromatography/tandem mass spectrometry (UPLC-MS/MS).

The sample extracts were analyzed using a UPLC–electrospray ionization (ESI)-MS/MS system. The analytical conditions were as follows: UPLC: column, Agilent SB-C18 (1.8 µm, 2.1 mm × 100 mm); the mobile phase consisted of solvent A (pure water with 0.1% formic acid) and solvent B (acetonitrile with 0.1% formic acid). Sample measurements were performed with a gradient program that employed the starting conditions of 95% solvent A and 5% solvent B. Within 9 min, a linear gradient to 5% solvent A and 95% solvent B was achieved and held for 1 min. Subsequently, the composition was adjusted to 95% solvent A and 5.0% solvent B within 1.1 min and held for 2.9 min. The flow velocity was set at 0.35 mL/min. The column oven was set to 40 °C. The injection volume used was 4 μL. The effluent was alternatively connected to an ESI-triple quadrupole-linear ion trap (QTRAP)-MS. The ESI source operation parameters were source temperature, 550 °C; ion spray voltage, 5500 V (positive ion mode)/−4500 V (negative ion mode). Ion source gas I (GSI), gas II (GSII), and curtain gas (CUR) pressures were 50, 60, and 25 psi, respectively. The collision-activated dissociation (CAD) was high. Triple quadrupole (QQQ) scans were acquired as MRM experiments with collision gas (nitrogen) set to medium. Declustering potential (DP) and collision energy (CE) for individual MRM transitions were performed with further DP and CE optimization. A specific set of MRM transitions was monitored for each period according to the metabolites eluted within this period.

According to the method of Chen et al. [21], the metabolites were analyzed qualitatively and quantitatively based on the fragment mode, retention time, MZ, MWDB v2.0 database (metware Biotechnology Co., Ltd., Wuhan, China), and public database standards built by Wuhan metware Biotechnology Co., Ltd. After obtaining the metabolic spectrum of different samples, the peak area of the mass spectrum peak of the substance was integrated. At the same time, the mass spectrum peak of the same metabolite in different samples was integrated and corrected. The mass spectrum data were processed by the software analvst 1.6.3. The peak area of each chromatographic peak of the metabolite represents the relative content of the corresponding substance.

Qualitative and quantitative analyses of metabolites were performed according to the methods of Zhao et al. [22]. Qualitative analysis of primary and secondary metabolites was carried out by comparison of the accurate fragmentation patterns, retention times (RT), and accurate *m*/*z* values based on the self-compiles MWDB database (Metware Biotechnology Co., Ltd., Wuhan, China; https://www.metware.cn accessed on 16 September 2024) and other public metabolite databases, namely Mass Bank (http://www.massbank.jp/ accessed on 16 September 2024), KNAPSAcK (http://www.knapsackfamily.com/KNApSAcK/ accessed on 16 September 2024), HMDB (http://www.hmdb.ca/ accessed on 16 September 2024) and ChemBank (https://data.broadinstitute.org/chembank/ accessed on 16 September 2024), PubChem (https://pubchemblog.ncbi.nlm.nih.gov/ accessed on 16 September 2024), NIST chemistry Webbook (http://webbook.nist.gov/ accessed on 16 September 2024), and METLIN (https://metlin.scripps.edu/ accessed on 16 September 2024). Finally, the chromatographic peak area was used to determine the relative metabolite contents.

### 2.5. RNA-Seq and Identification of Differentially Expressed Genes

The RNA from the leaves collected at three different developmental stages was sequenced. Total RNA extraction and complementary DNA (cDNA) library construction were conducted using the MWDB database (MetWare). RNA degradation and contamination were monitored on 1% agarose gels. RNA purity was checked using a NanoPhotometer^®^ spectrophotometer (IMPLEN, Westlake Village, CA, USA). RNA concentration was measured using a Qubit^®^ RNA Assay Kit and a Qubit^®^2.0 Fluorometer (Life Technologies, Carlsbad, CA, USA). RNA integrity was assessed using an RNA Nano 6000 Assay Kit and a Bioanalyzer 2100 system (Agilent Technologies, Santa Clara, CA, USA). Freeze-dried leaf samples were used for this analysis.

A total of 1 µg of RNA from each sample was used as input material. Sequencing libraries were generated using an NEBNext^®^ Ultra^TM^ RNALibrary Prep Kit for Illumina^®^ (NEB, Ipswich, MA, USA) following the manufacturer’s recommendations; index codes were added to attribute sequences for each sample. Finally, PCR products were purified using the Agencourt AMPure XP Kit (Beckman Coulter Trading (China) Co., Ltd., Shanghai, China), (AMPure XP system), and library quality was assessed using an Agilent Bioanalyzer 2100 system. The clustering of the index-coded samples was performed using a cBot Cluster Generation System with a TruSeq PE Cluster Kit v3-cBot-HS (Illumina) according to the manufacturer’s instructions. After cluster generation, the library preparations were sequenced on an Illumina platform, and 150 bp paired-end reads were generated. The RNA-seq data have been deposited in the NCBI Sequence Read Archive (NCBIvSRA) under accession number PRJNA1051355.

### 2.6. Evaluation of Gene Expression Using Quantitative Reverse Transcription-PCR (qRT-PCR)

The relative expression of nine DEGs identified in transcriptome analysis was evaluated using qRT-PCR to validate the transcriptome data. Firstly, single-stranded cDNA was synthesized from total RNA. RNA extraction and cDNA synthesis were conducted using the Tiangen total RNA extraction kit (Tiangen, Beijing, China) and PrimeScript RTreagent Kit with gDNA Eraser (TaKaRa, Kyoto, Japan). Nine gene-specific primers were designed using Primer Premier v5.0 (Premier Biosoft, Palo Alto, CA, USA) (Table S1). qRT-PCR was performed using three biological replicates on the Bio Rad CFX96 real-time PCR detection system (Hercules, CA, USA). qRT-PCR tests were carried out with the TaKaRa SYBR Green Mix kit (TaKaRa, Beijing, China) using the ABI 7500Fast Real-Time Detection System. PCR amplification was performed in a 20 µL volume containing 10 µL, 2× SYBR Primix ExTaq, 0.8 µL upstream primer, 0.8 µL downstream primer, 0.4 µL Rox-Reference dye, 2 µL cDNA template, and 6 µL ddl H_2_O, under a standard PCR program of 95 °C for 30 s; 40 cycles at 95 °C for 5 s; 60 °C for 35 s, 95 °C for 15 s; and 60 °C for 1 min, followed by 95 °C for 15 s. Quantitative expression analysis was conducted using the housekeeping gene Cluster-466325.5 of *D. cercidifolius* as a reference gene

### 2.7. Statistical Analysis

Statistical analyses of leaf chemical composition were conducted using IBM SPSS 22.0 software statistics (IBM Corp., Armonk, NY, USA). Data were analyzed using one-way Analysis of Variance (ANOVA) and Pearson’s correlation tests at a 5% significance level [22]. Based on the Metware cloud platform, principal component analysis (PCA), hierarchical cluster analysis (HCA), and partial least squares discriminant analysis (PLS-DA) were performed to screen differential metabolites. The metabolites with VIP > 1, *p*-value/FDR ≤ 0.05, and |log2FC| > 1 were considered differentially accumulated metabolites (DAMs). Kyoto Encyclopedia of Genes and Genomes (KEGG) pathway analysis was conducted to annotate metabolites using the KEGG Orthology software (http://kobas.cbi.pku.edu.cn/ (accessed on 17 November 2022)). Genes exhibiting differences in expression were analyzed using the DESeq2 (v1.16.1) software. Genes with a fold change ≥ 2 and false discovery rate < 0.05 were identified as DEGs. Furthermore, DEGs were described in detail based on KEGG pathways.

The differences in metabolites and gene expression at different developmental stages were tested using one-way paired ANOVA in PASW Statistics 18 (SPSS Inc., Chicago, IL, USA); *p* < 0.05 indicated significance.

## 3. Results

### 3.1. Differences in the Chemical Composition of Leaves During Different Developmental Stages

**Figure 1:**
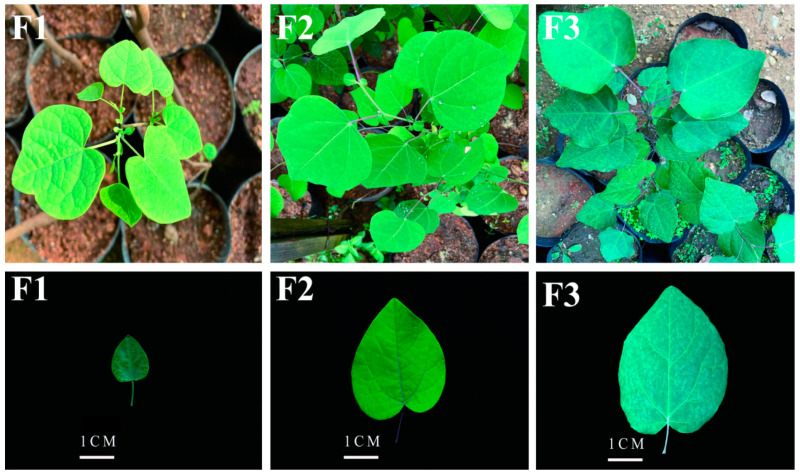
Differences in leaf morphology of *D. cercidifolius* var. longipes at different developmental stages. F1, young leaves (leaves unfolded, but the internal structure is not fully mature); F2, near-mature leaves (the leaf area just reaches the maximum after leaf unfolding, and the internal structure of leaves tends to be mature); and F3, mature leaves (leaves are fully mature).

We compared the chemical compositions of different developmental stages in the leaves of *D. cercidifolius* to understand the dynamic changes in primary and secondary metabolites during leaf development (Figure 1, Table 1). According to Table 1, as the leaves mature gradually, the contents of free amino acids, soluble protein, and soluble sugar decrease gradually, and the differences between different stages are significant (*p* < 0.05). The total flavonoid contents first decreased, then increased, and were the highest in F3. The trends of total phenolic content were consistent with those of total flavonoid contents; however, there was no significant difference in total phenolic content between F1 and F3. The total alkaloid contents first increased and then decreased during development, with F3 having the lowest contents. The total triterpenoid content trends were consistent with the total alkaloid content trends; however, there were no significant differences between F1 and F3.

### 3.2. Differential Metabolite Analysis During Leaf Development

**Figure 2:**
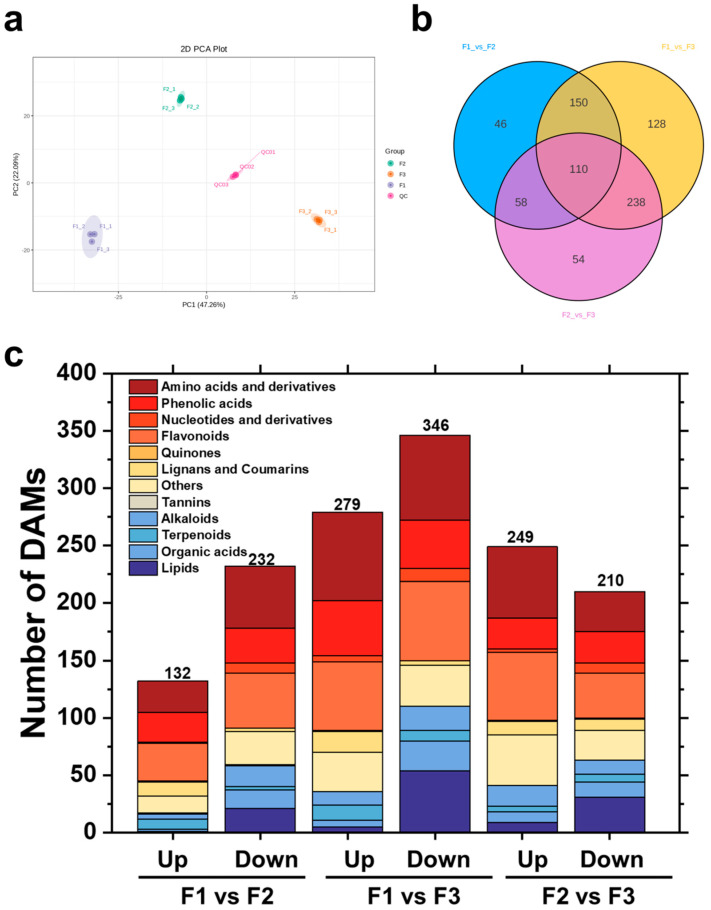
Analysis of leaf metabolites at different developmental stages. (**a**) Principal component analysis of leaves at three stages; (**b**) Venn diagram of differential metabolites in the three comparison groups; (**c**) up- and downregulation of a single comparison group of differential metabolites by type. F1, young leaves (leaves unfolded, but the internal structure is not fully mature); F2, near-mature leaves (the leaf area just reaches the maximum after leaf unfolding, and the internal structure of leaves tends to be mature); and F3, mature leaves (leaves are fully mature).

The data obtained from UPLC/ESI-QTRAP-MS/MS were analyzed to detect metabolite changes during different leaf developmental stages. The principal component analysis and correlation analysis results showed obvious separation among the leaves of the three stages (Figure 2a). Biological replicates were consistent and highly correlated. This result ensured that the follow-up analysis was reliable (Figure S1). In total, 1254 metabolites were detected in the leaves. The Venn diagram showed that approximately 62.51% of metabolites exhibited significant differences during leaf development in *D. cercidifolius* (784 DAMs) (Figure 2b). Among the three comparison groups, there were 110 common differential metabolites, indicating significant differences in metabolites during different stages of leaf development in the long-stalked flowering plant, which is consistent with the results of PCA. It is worth noting that flavonoids, amino acids and derivatives, phenolic acids, and lipids account for a large proportion of 71.82% of the 110 common differential metabolites (Table S2). A total of 364 DAMs were detected between F1 and F2, including 132 upregulated DAMs and 232 downregulated DAMs; 715 DAMs were detected between F1 and F3, out of which 279 were upregulated, and 346 were downregulated; and 459 DAMs were detected between F2 and F3, including 249 upregulated DAMs and 210 downregulated DAMs (Figure 2c).

To better understand the differences in metabolites in different developmental stages of the leaves of *D. cercidifolius*, the paired comparison group PLS-DA model was established to screen the different metabolites (Figure S2). The results showed that the Q2 values of each comparison group were greater than 0.5, indicating that the model has certain reliability and good predictive reliability, which can be used to analyze differential metabolites.

### 3.3. Screening of DAMs and KEGG Annotation

**Figure 3:**
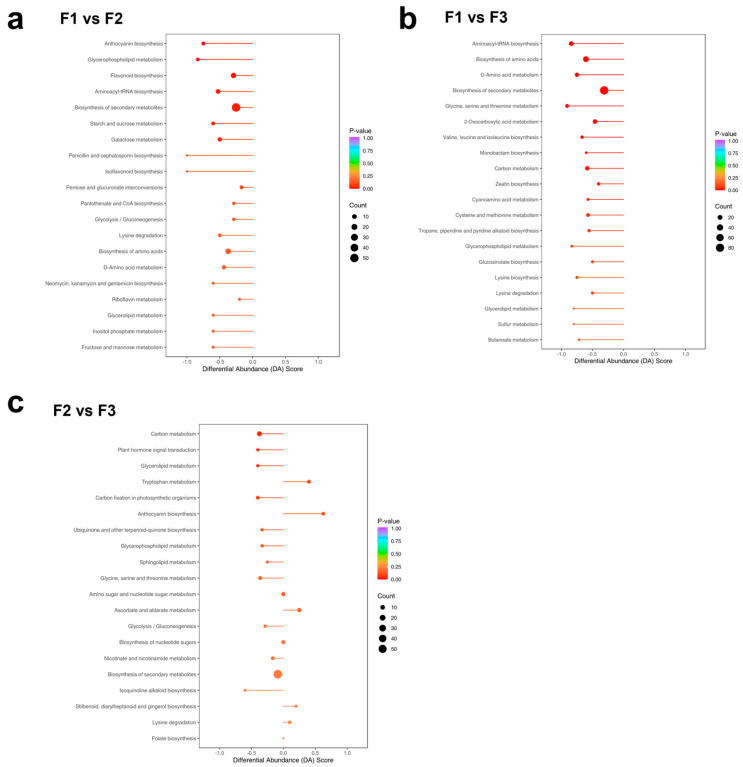
Kyoto Encyclopedia of Genes and Genomes (KEGG) analysis of leaf metabolites at three developmental stages. (**a**) F1 and F2 leaves; (**b**) F1 and F3 leaves; and (**c**) F2 and F3 leaves. F1, young leaves (leaves unfolded, but the internal structure is not fully mature); F2, near-mature leaves (the leaf area just reaches the maximum after leaf unfolding, and the internal structure of leaves tends to be mature); and F3, mature leaves (leaves are fully mature).

The differential metabolites were annotated across the three stages of development to understand the function of DAMs. KEGG enrichment analysis showed that differential metabolites were concentrated in anthocyanin synthesis, C metabolism, and flavonoid biosynthesis (Figure 3). Metabolites in the anthocyanin synthesis pathway were downregulated in F1, upregulated in F2, and finally became similar to F1 in F3; there was no significant difference in the comparison between F1 and F3. Metabolites related to the C metabolic pathway and amino acid biosynthesis were significantly downregulated in F2; there was no significant difference between F1 and F2. The amino acid-related synthesis pathway was downregulated in F2. In F3, amino acid metabolism pathways were significantly downregulated, excluding the tryptophan metabolism pathway. Conversely, the flavonoid metabolism pathway was downregulated in F1 leaves and upregulated slightly in F2; there was no significant difference between F1 and F3 in flavonoid metabolism.

**Figure 4:**
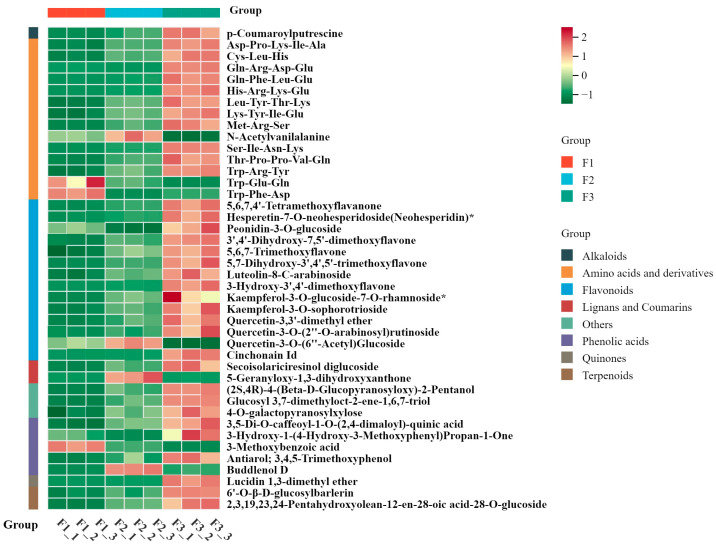
Heatmap of significant differences in common differential metabolites at three developmental stages. The green and red segments represent low and high abundance of metabolites, respectively. * represents that the substance has isomers.

Based on fold changes ≥ 2 or ≤0.5 and *p*-value/FDR ≤ 0.05, a total of 42 common differential metabolites with significant changes were identified (Table S2), mainly belonging to flavonoids (14, 33.3%), phenolic acids (5, 11.9%), others (3, 7.1%), amino acids and derivatives (14, 33.3%), lignans and coumarins (2, 4.8%), alkaloids (1, 2.4%), terpenoids (2, 4.8%), and quinones (1, 2.4%). Thirty-five metabolites were significantly higher in F2 and F3 than in F1, with only four compounds, N-acetylvanillanine, Trp-Glu-Gln, Trp-Phe-Asp, and 3-methoxybenzoic acid, significantly lower than in F1. Additionally, N-acetylvanillanine, Quercetin-3-O-(6″-Acetyl) Glucoside, 5-Geranyloxy-1,3-dihydroxyxanthone, and Buddenol D were significantly higher in F2 than in F1 and F3(Figure 4).

### 3.4. Overview of Transcriptome Sequencing

**Figure 5:**
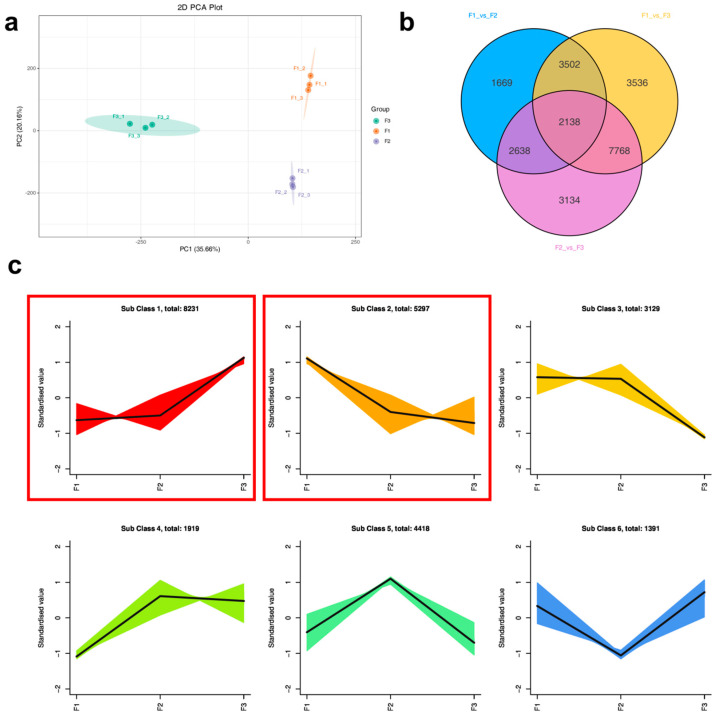
Transcriptome analysis of the three stages of leaf development. (**a**) Principal component analysis of leaves at the three stages; (**b**) Venn diagram of differential genes; (**c**) expression trends of differentially expressed genes. Each cluster represents a changing pattern. For example, genes in cluster 1 are defined as genes whose expression gradually increases with leaf development, F3 > F2 > F1 (gene expression). F1, young leaves (leaves unfolded, but the internal structure is not fully mature); F2, near-mature leaves (the leaf area just reaches the maximum after leaf unfolding, and the internal structure of leaves tends to be mature); and F3, mature leaves (leaves are fully mature).

To further verify the results from the metabolome, transcriptomic measurements were conducted on leaves at three developmental stages. The principal component analysis results showed that leaves from the three stages were separated from each other (Figure 5a). Based on a Venn diagram, there were 9947 differential genes between F1 and F2, 16,944 differential genes between F1 and F3, and 15,678 differential genes between F2 and F3; among them, 2138 differential genes were common among the three stages (Figure 5b). Among the differential genes between F1 and F2, there were 4596 downregulated genes and 5351 upregulated genes. Among the differential genes between the F2 and F3 leaves, there were 3638 downregulated genes and 8306 upregulated genes. Among the differential genes between F1 and F3, there were 8485 downregulated genes and 7193 upregulated genes (Figure S3). K-means clustering was used to draw the expression maps of differential genes. The results showed that 8231 genes in the first cluster exhibited overall upregulation, and 5297 genes in the second cluster exhibited overall downregulation (Figure 5c).

### 3.5. KEGG Pathway Analysis of DEGs

**Figure 6:**
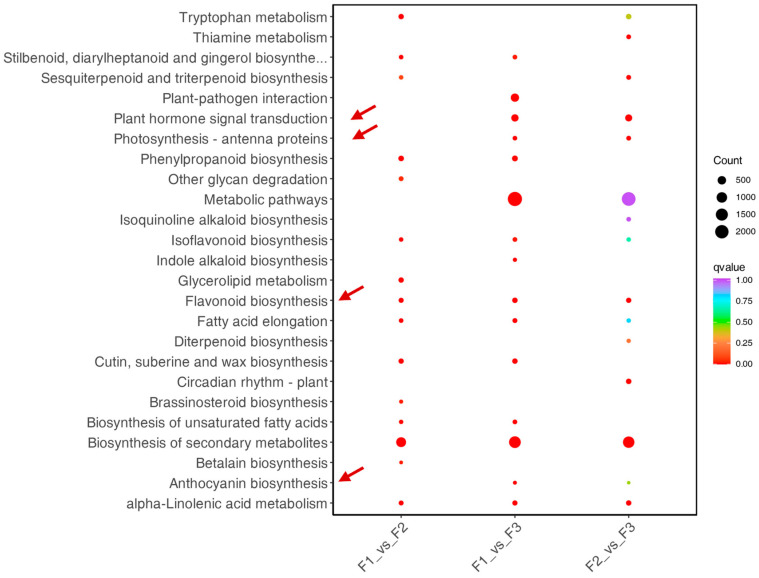
KEGG analysis of leaf transcriptome data from the three stages. F1, young leaves (leaves unfolded, but the internal structure is not fully mature); F2, near-mature leaves (the leaf area just reaches the maximum after leaf unfolding, and the internal structure of leaves tends to be mature); and F3, mature leaves (leaves are fully mature).

A KEGG pathway enrichment analysis was performed to understand the expression of DEGs. Consistent with the metabolite data, differential genes were enriched in photosynthesis, energy metabolism, anthocyanin synthesis, and flavonoid synthesis pathways. Flavonoid synthesis showed a higher Q-value in F2 (Figure 6 and Figure S4), while photosynthesis-antenna protein and flavonoid synthesis showed a higher Q-value in F2 and F3 (Figure 5). High Q-values mainly enriched the differential genes between F1 and F3 in pathways such as anthocyanin and flavonoid synthesis (Figure 5). To clarify the overall status of differential gene enrichment among the three stages, the differential genes in each stage were combined for enrichment analysis, including the photosynthesis-antenna protein, flavonoid synthesis, plant hormone signal transduction, and anthocyanin synthesis pathways. The other channels all had a higher Q-value.

### 3.6. Combined Transcriptomic and Metabolomic Analysis

**Figure 7:**
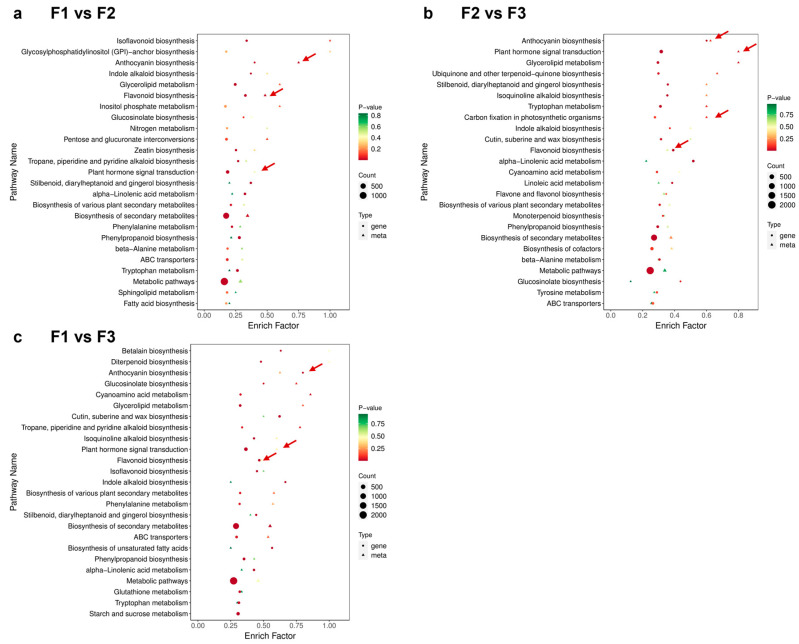
Joint analysis of differential metabolites and differential genes. F1, young leaves (leaves unfolded, but the internal structure is not fully mature); F2, near-mature leaves (the leaf area just reaches the maximum after leaf unfolding, and the internal structure of leaves tends to be mature); and F3, mature leaves (leaves are fully mature).

A joint analysis was conducted to better understand the relationship between the transcriptome and metabolome results during leaf development (Figure 7). Combined with the phenomena related to leaf development, such as substance accumulation and color changes, the present study investigated the anthocyanin and flavonoid synthesis pathways through joint analysis. The two pathways were enriched significantly in all three groups; the results are highly consistent with transcriptome and metabolome analysis results during leaf development. The present study also focused on the biosynthesis pathway of carbon dioxide fixation in photosynthesis, as *D. cercidifolius* is a shade-loving plant. Plant hormone signal transduction pathways are also significantly enriched.

### 3.7. Analysis of Gene Expression Through qRT-PCR

**Figure 8:**
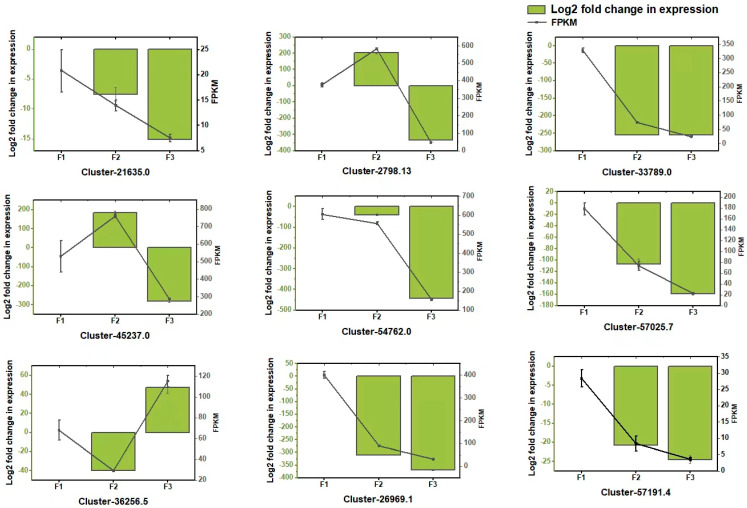
qRT-PCR validation of differentially expressed genes. Values are presented as means ± standard deviation of three independent measurements.

To validate the transcriptome data, nine DEGs related to flavonoid and anthocyanin biosynthesis were selected for expression analysis in F1, F2, and F3 using qRT-PCR (Figure 8). They are as follows: Anthocyanidin 5,3-O-glucosyltransferase (Cluster-21635.0), Chalcone–flavanone isomerase (Cluster-2798.13), cytochrome P450 (Cluster-33789.0), Chalcone synthase (Cluster-45237.0), Flavanone-3-hydroxylase (Cluster-54762.0), Cinnamyl alcohol dehydrogenase (Cluster-57025.7), UDP-glycosyltransferase (Cluster-36256.5), Flavone synthase (Cluster-26969.1), and Chalcone isomerase (Cluster-57191.4). The expression profiles of the candidate genes were consistent with the RNA-seq results, indicating that the RNA-seq data obtained through Illumina sequencing and qRT-PCR are consistent.

## 4. Discussion

The growth and development of leaves play important functions in the life history of plants. If their growth and development are disrupted, it typically affects their overall health [23]. Therefore, researchers have been investigating the leaf development processes of different plants. In such studies, using gene expression data to explain the mechanisms of metabolic activity is usually a major approach [24]. The process of change in *D. cercidifolius* leaves is also shown here. The results of transcriptomic and metabolomic studies during the development of *D. cercidifolius* leaves showed that the DAMs and DEGs were both enriched in pathways related to photosynthesis, C metabolism, anthocyanin synthesis, and flavonoid synthesis. The importance of the pathways in leaf development has been reported in many plants [25,26]. However, such information can only describe life activities within plants but cannot reveal the ecological adaptation significance of the activities; consequently, it is necessary to discuss such life activities in the context of environmental change.

Carbon metabolism is the most basic metabolic process in plants. Its metabolic intensity and dynamic changes affect the growth and development of plants. Light intensity is generally believed to positively affect plants’ accumulation of primary metabolites. A low-light environment during grain filling can increase amino acid content during rice formation [27]. Under the shading condition, the protein content accumulation in soybeans increased [28]. Leaf buds mature and unfold in *D. cercidifolius* in spring [3]. At this time, the natural distribution area’s temperature, light, and humidity (China) benefit leaf development, and the leaves’ free amino acid and soluble sugar contents are the highest. The high accumulation levels of these substances may be in preparation for subsequent critical life activities, such as leaf area expansion and color change. At the second stage of development (near-ripe leaves, F2), the distribution area of *D. cercidifolius* is in the stage of transition from spring to summer, and the shade-loving plant, *D. cercidifolius*, is about to enter summer, which is associated with high light intensity. The increase in light intensity is harmful to shade-loving plants.

Plants will adopt some protective strategies to avoid damage from excessive sunlight. The accumulation of flavonoids in the leaves of *D. cercidifolius* can enhance shade tolerance under light stress. Primary metabolites are precursors for forming secondary metabolites, providing them with synthetic substances and energy. At the same time, the accumulation of secondary metabolites is also closely related to the light intensity. The biosynthesis of flavonoids and phenols needs light or is regulated by light. The biosynthesis of flavonoids is completely dependent on light, and their biosynthesis rate is related to light intensity and density. The change in light intensity significantly impacts the production and accumulation of total flavonoids and phenols in plants [29]. *Zingiber officinale* Rosco accumulated more flavonoids in its leaves under 60% shading treatment, and tannins also accumulated in large amounts [30]. In *Camellia sinensis*, shading treatment can promote the accumulation of phenolic acids in tea [31]. Such substances enhance the antioxidant capacity of leaves and minimize photooxidation damage. However, accumulating such substances influences plant leaves’ C metabolism and fixation ability.

Light intensity significantly affects the photosynthesis of *D. cercidifolius* and then affects the primary metabolism, secondary metabolism accumulation, and yield formation of *D. cercidifolius* [32]. Photosynthesis in plant leaves depends on light and is closely related to antioxidant enzyme activity and endogenous hormone content in leaves. The interaction of light and hormones is important in regulating plant growth and development [33]. Gibberellin (GA), abscisic acid (ABA) [34], and indole-3-acetic acid (IAA) [35] are the main hormones involved in the regulation of axillary bud growth. Hormones are also involved in the regulation of photosynthesis in plants. The interaction of light and hormone signal transduction pathways plays an important role in regulating plant morphogenesis. The enrichment analysis of differential metabolites and genes in the three stages of leaf development in *D. cercidifolius* showed that the plant hormone signal transduction pathway was also significantly enriched. Plants are stimulated by light, producing bioactive small molecule compounds that regulate physiological response hormones, inducing gene transcription in a variety of ways, and synergistically affecting seed germination, growth, leaf and root development, chlorophyll synthesis, and decomposition of plants, which plays an important role in regulating plant growth and development. Some flavonoids can control the auxin content by controlling the activity of indoleacetic acid oxidase, ultimately affecting plants’ growth and development [36]. It could be inferred that the photosynthesis antenna protein pathway, flavonoid synthesis pathways, and plant hormone signal transduction pathway all play an important role in the leaf development process of *D. cercidifolius*.

Anthocyanin accumulation in apple leaves decreases C sequestration and photosynthetic capacity [37], as well as in *D. cercidifolius*. Compared with the early developmental stage (F1) and mature leaf stage (F3), anthocyanin accumulation was the highest, and photosynthetic capacity and C fixation capacity were the lowest in the metaphase stage (F2) because of the lowest expression levels of related genes at the metaphase stage. An increase in the transformation of plant secondary metabolites would consume a lot of nutrients, and the decrease in photosynthetic capacity may make the potential energy input and energy output unbalanced, leading to impairment of subsequent life activities [38].

When the leaves matured (F3), the metabolites on the anthocyanin metabolic pathway in the leaves were downregulated compared to within the middle stage of development (F2), and C metabolism capacity was enhanced. The two changes may be interlinked. The stage falls in the summer, with high temperatures and strong light; however, the downregulation of gene expression in anthocyanin the biosynthesis pathway in the leaves of *D. cercidifolius* implies a decline in light protection capacity, which does not match with the environmental change. Admittedly, the total flavonoid contents in leaves were the highest in the mature stage, which implies that leaves have strong photoprotection potential. In addition, continuous anthocyanin synthesis and low photosynthesis make it challenging to maintain tree development.

In contrast, enhancing light and capacity can compensate for the lack of energy supply. Therefore, this change in mature leaves may be a manifestation of adaptability [39]; that is, the photoprotection ability is enhanced by flavonoid and anthocyanin accumulation in the early stage, and the development is maintained by enhanced light, and the photoprotection capacity in the later stage. Through physiological adaptability, *D. cercidifolius* can synthesize sufficient nutrients before flower bud differentiation in July for subsequent plant reproduction and survival.

Due to the increase in the intensity of human activities, including urbanization and land use, many plants are being exposed to sunlight, including *D. cercidifolius*. The increase in light intensity is likely to make it more expensive physiologically for *D. cercidifolius* to survive; this has a destructive effect on *D. cercidifolius*, which has sparse (small number) leaves, as the total leaf area is critical for energy fixation efficiency. To achieve light protection, the accumulation times of anthocyanins or flavonoids may be increased, and photosynthetic capacity enhancement may be delayed. Conversely, photoperiod regulates flowering mainly, although light intensity is also affected. Therefore, the time node of flower development of *D. cercidifolius* may not change. A delay in an increase in photosynthetic as anthesis remains the same leads to an imbalance in the synthesis and distribution of energy, thus undermining the robustness of the plant life cycle, which could explain why the fruit-set rate of the natural *D. cercidifolius* population is extremely low, the seeds are poorly developed, and germination rate is not high, which may also be a reason why *D. cercidifolius* is endangered. Therefore, shading measures are critical for the conservation of *D. cercidifolius*.

## 5. Conclusions

*D. cercidifolius* is a valuable tree species with ornamental and research value. In its natural state, it accumulates large amounts of primary metabolites in its newly developed leaves (F1). Near-mature leaves (F2) will increase metabolites’ contents and gene expression in the anthocyanin synthesis and flavonoid metabolism pathways and transform high amounts of primary metabolites into secondary metabolites to cope with strong light in summer. Moreover, photosynthetic capacity will decrease at this stage (the overall decrease in gene expression involved in C metabolism and C fixation pathways). The photosynthetic capacity of leaves recovers after maturity (F3), and flavonoid contents were the highest. The observations suggest that the leaves have the strongest light protection and photosynthetic capacity after maturity. Like many plants, *D. cercidifolius* achieves light protection while regulating C fixation and C metabolism at different leaf development stages to achieve stable growth and development; this is an adaptive strategy for shade-loving plants. In addition, the photosynthesis antenna protein pathway, flavonoid synthesis pathways, and plant hormone signal transduction pathway work together to affect leaf development in transcriptome data. In the wake of habitat destruction activities of human activities, *D. cercidifolius* light stress is increasing; this may lead to enhanced transformation of secondary metabolites or delay the recovery of photosynthetic capacity, undermining energy fixation and distribution in plants. Therefore, to protect this valuable plant, it is necessary to erect shades or cultivate *D. cercidifolius* in shaded environments.

## Figures and Tables

**Table 1:** Chemical composition analysis of leaves of *D. cercidifolius* var. *longipes* at different development stages. Different letters represent significant differences among the different development stages. (*p* < 0.05).

Different Development Stages	F1	F2	F3
Free amino acids (FW mg/g)	2.32 ± 0.03 a	0.82 ± 0.00 b	0.36 ± 0.01 c
Soluble protein content (FW mg/g)	4.65 ± 0.19 a	2.03 ± 0.05 b	1.45 ± 0.04 c
Soluble sugar content (FW mg/g)	56.70 ± 2.36 a	43.58 ± 1.02 c	40.40 ± 1.92 b
Total flavonoids content (FW mg/g)	21.34 ± 0.99 b	18.17 ± 0.58 c	23.21 ± 0.52 a
Total phenols content (FW mg/g)	14.52 ± 0.14 a	11.04 ± 0.48 b	13.86 ± 0.41 a
Total alkaloids content (FW mg/g)	14.01 ± 0.55 b	18.97 ± 0.90 a	10.70 ± 0.33 c
Total triterpenoids content (DW mg/g)	55.46 ± 2.13 a	62.19 ± 3.08 b	55.20 ± 1.29 a

## Data Availability

The datasets generated during and/or analyzed during the current study are available from the corresponding author upon reasonable request. The RNA-seq data have been uploaded to the Gene Expression Omnibus database (GEO) with the accession number GSE254419. Metabolomic data have been deposited in MetaboLights with the accession number MTBLS9466.

## Supplementary Materials

The following supporting information can be downloaded at https://www.mdpi.com/article/10.3390/metabo14120654/s1. Table S1: primers for qRT-PCR; Table S2: Significant common differential metabolites at three developmental stages; Figure S1: Correlation analysis between leaf samples at three stages; Figure S2: PLS-DA analysis of leaves at different development stages; Figure S3: Trends of differential gene expression within the comparison groups; Figure S4: Kyoto Encyclopedia of Genes and Genomes (KEGG) analysis of leaf transcriptome at three developmental stages. (a) F1 and F2 leaves; (b) F1 and F3 leaves; and (c) F2 and F3 leaves. F1, young leaves (leaves unfolded, but the internal structure is not fully mature); F2, near-mature leaves (the leaf area just reaches the maximum after leaf unfolding, and the internal structure of leaves tends to be mature); and F3, mature leaves (leaves are fully mature).
