# Supplementary figures and images for: Integrated Transcriptomic and Metabolomic Analysis Reveals Possible Molecular Mechanisms of Leaf Growth and Development in Disanthus cercidifolius var. longipes
Source: Metabolites. 2024 Nov 25;14(12):654. doi: 10.3390/metabo14120654 (PMC11678885; doi:10.3390/metabo14120654)

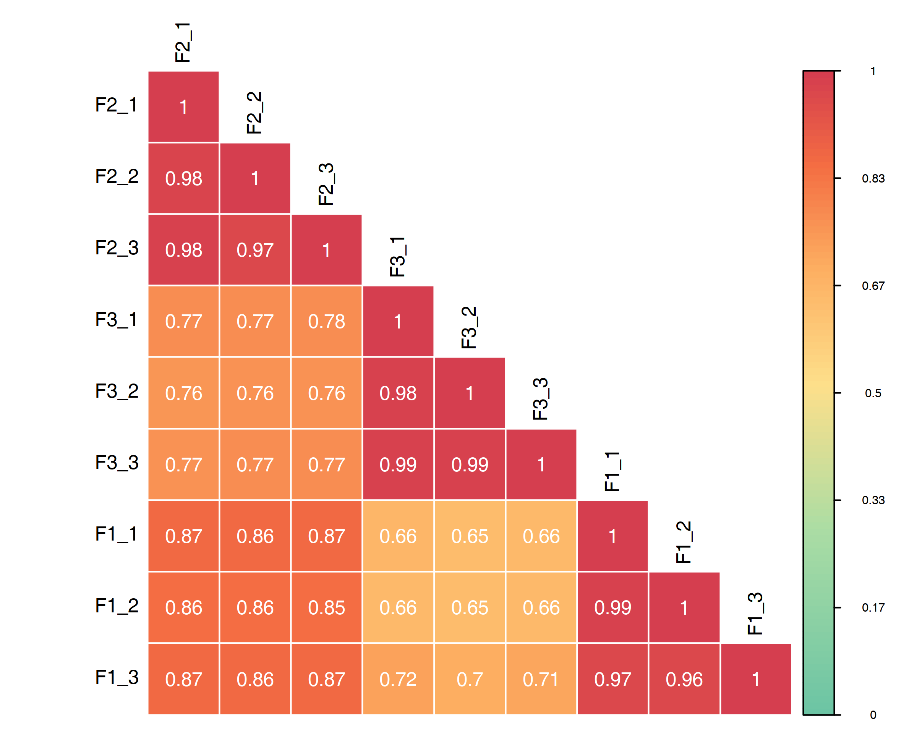

Supplement: Supplementary file 1 [file metabolites-14-00654-s001.zip › Figure S1.png]

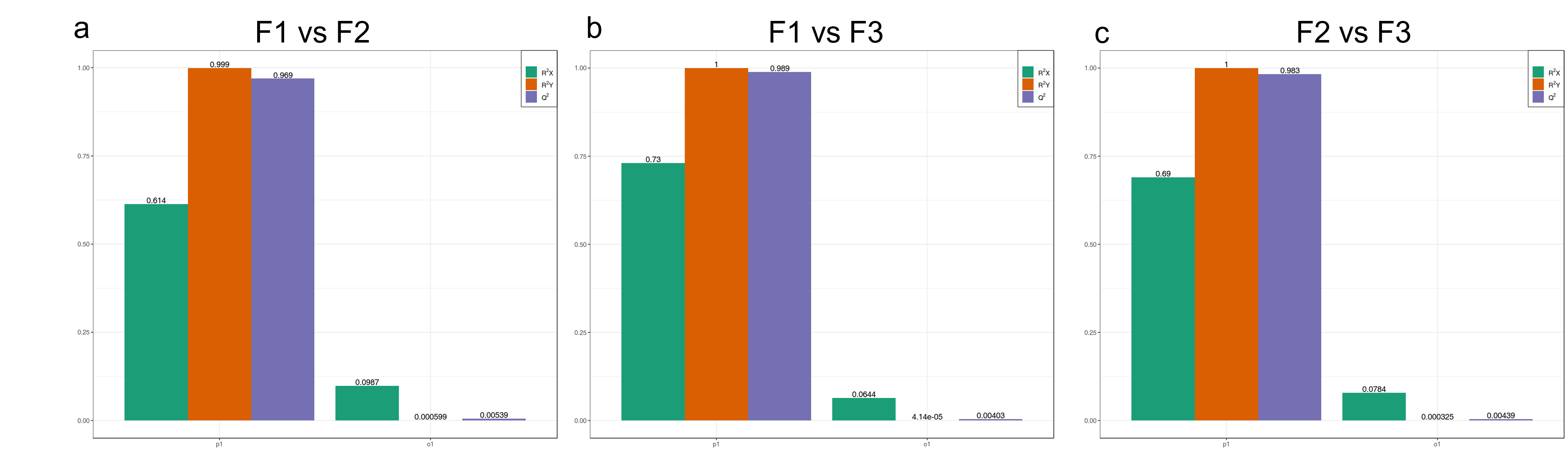

Supplement: Supplementary file 1 [file metabolites-14-00654-s001.zip › Figure S2.png]

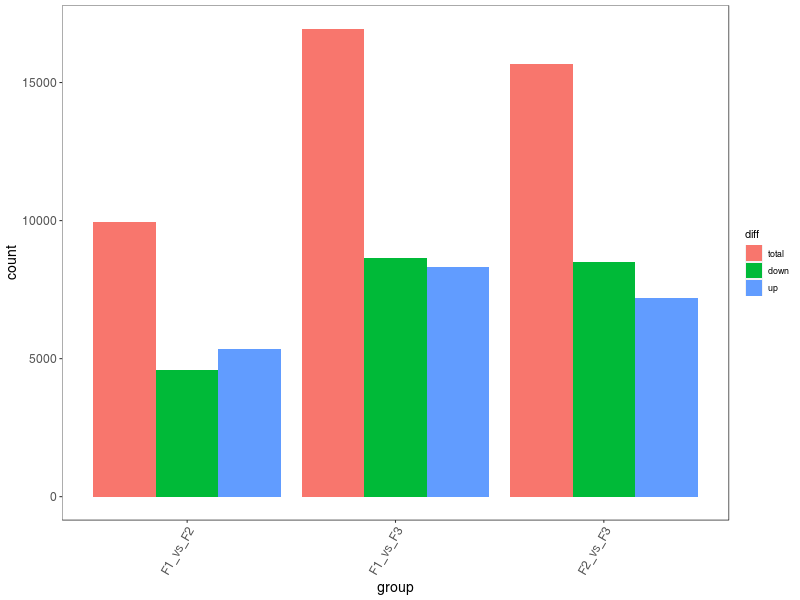

Supplement: Supplementary file 1 [file metabolites-14-00654-s001.zip › Figure S3.png]

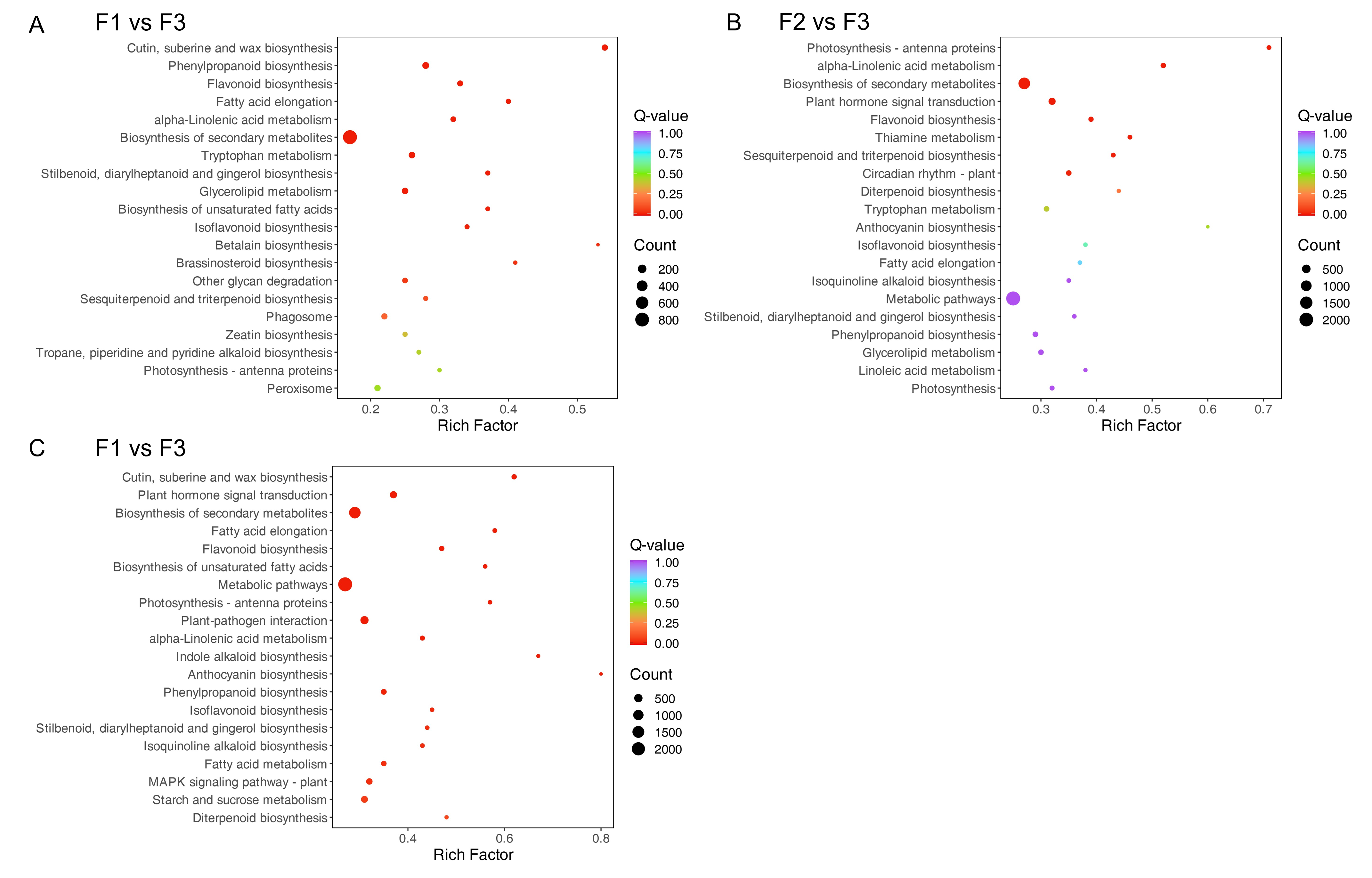

Supplement: Supplementary file 1 [file metabolites-14-00654-s001.zip › Figure S4.tif]
